# Small Extracellular Vesicles Derived from Human Chorionic MSCs as Modern Perspective towards Cell-Free Therapy

**DOI:** 10.3390/ijms222413581

**Published:** 2021-12-18

**Authors:** Jana Janockova, Jana Matejova, Marko Moravek, Lucia Homolova, Lucia Slovinska, Alena Nagyova, Dmytro Rak, Marian Sedlak, Denisa Harvanova, Timea Spakova, Jan Rosocha

**Affiliations:** 1 Associated Tissue Bank, Faculty of Medicine, P. J. Safarik University and L. Pasteur University Hospital in Kosice, Tr. SNP 1, 04011 Kosice, Slovakia; jana.matejova@upjs.sk (J.M.); marko.moravek@student.upjs.sk (M.M.); lucia.homolova@upjs.sk (L.H.); lucia.slovinska@upjs.sk (L.S.); denisa.harvanova@upjs.sk (D.H.); timea.spakova@upjs.sk (T.S.); jan.rosocha@upjs.sk (J.R.); 2 Department of Gynaecology and Obstetrics, Faculty of Medicine, P. J. Safarik University and L. Pasteur University Hospital in Kosice, Tr. SNP 1, 04011 Kosice, Slovakia; alena.nagyova@upjs.sk; 3 Institute of Experimental Physics, Slovak Academy of Sciences, Watsonova 47, 04001 Kosice, Slovakia; rak@saske.sk (D.R.); marsed@saske.sk (M.S.)

**Keywords:** extracellular vesicles, small EVs, chorion, mesenchymal stem cells, cell-free therapy

## Abstract

Mesenchymal stem cells (MSCs) are of great interest to scientists due to their application in cell therapy of many diseases, as well as regenerative medicine and tissue engineering. Recently, there has been growing evidence surrounding the research based on extracellular vesicles (EVs), especially small EVs (sEVs)/exosomes derived from MSCs. EVs/exosomes can be secreted by almost all cell types and various types of EVs show multiple functions. In addition, MSCs-derived exosomes have similar characteristics and biological activities to MSCs and their therapeutic applications are considered as a safe strategy in cell-free therapy. The aim of this study was the characterization of MSCs isolated from the chorion (CHo-MSCs) of human full-term placenta, as well as the isolation and analysis of small EVs obtained from these cells. Accordingly, in this study, the ability of small EVs’ uptake is indicated by synovial fibroblasts, osteoblasts and periosteum-derived MSCs. Improvement in the understanding of the structure, characteristics, mechanism of action and potential application of MSCs-derived small EVs can provide new insight into improved therapeutic strategies.

## 1. Introduction

Mesenchymal stem cells (MSCs) are multipotent stem cells firstly isolated from adult bone marrow [1,2] and have become studied and widely used in cell-based therapy over the past 30 years [3]. MSCs of different origin are characterized with several equal features, but also with biological differences responsible for their distinct clinical properties and potential application in cellular therapy and tissue engineering, mostly to treat degenerative changes in joints, to reconstruct bones and cartilage, in cell transplantation, in cardiovascular diseases, in plastic surgeries and in aesthetic medicine [4,5,6,7,8].

There is an evidence that MSCs were successfully isolated from various sources, including bone marrow, adipose tissue, umbilical cord tissue, cord blood, placenta, amniotic fluid, synovial fluid, etc., [9,10]. The placenta is a very abundant source of MSCs considering its easy availability and noninvasive tissue collection, without causing ethical issues. Nowadays, MSCs isolation from placental membranes has been extensively examined because of exhibition of their different proliferative and differentiative potential, caused by the complex structures and functions of placenta [11]. The human placenta is a complex feto-maternal organ, which consists of the amniotic membrane, amniotic epithelium, chorionic membrane, chorionic trophoblast, chorion villi and decidua [12]. The amnion is the inside layer surrounding the fetus during pregnancy. Amniotic epithelial cells and amniotic MSCs could be extracted from the amnion. The chorion is situated outside and is attached to the decidua (maternal part of the placenta). Chorionic MSCs and chorionic trophoblastic cells could be extracted from the chorion [13,14]. Gonzalez et al. and Bacenkova et al. showed the superior differentiation of human chorionic-derived MSCs (CHo-MSCs) and their immunosuppressive and angiogenic potential in vitro [15,16]. Koo et al. confirmed that CHo-MSCs are able to express many pluripotent stem cell-specific genes and proliferate well during ex vivo expansion [17]. Yamahara et al. also indicated the secretion of angiogenic factors, including HGF, IGF-1, VEGF and bFGF by CHo-MSCs. Moreover, it was determined that transplantation of CHo-MSCs significantly increased blood flow and capillary density in a murine hindlimb ischemia model [18].

In general, MSCs are able to self-recover, can differentiate into many types of cells and participate in immunomodulation [19]. Their broad-ranging potential for the treatment of several diseases may be largely realized by paracrine factors presented by cytokines, chemokines and growth factors [20]. Recent studies confirmed the therapeutic effect of MSCs through their secretion of extracellular vesicles (EVs) [21], which are membranous structures derived from cells and which play an essential role in intercellular communication via transfer of bioactive proteins, lipids and RNAs. Through their heterogeneous composition, EVs are considered to be a potential source of circulating biomarkers of several diseases. Moreover, there is an evidence that EVs are promising candidates for cell-free regenerative medicine [22], due to their capability to affect cellular phenotype, proliferation and differentiation in a paracrine manner [23]. They have been tested in various animal models for human diseases (e.g., liver fibrosis [24], brain injury [25], bone defects [26], osteoarthritis [27], kidney injury [28], myocardial infarction [29], Alzheimer’s disease [30], wound healing and angiogenesis [31]) and it was detected that their functions are very similar to MSCs [22]. Both normal and pathological cells are able to release various types of EVs with different physiological properties, functions and compositions. The International Society of Extracellular Vesicles (ISEV) approved that “EVs is a generic term for particles secreted by cells that are delimited by a lipid bilayer and cannot replicate, i.e., do not contain a functional nucleus” [32]. Generally, EVs are classified based on their biogenesis and size into three main classes—apoptotic bodies (~50–2000 nm in diameter), microvesicles (~150–1000 nm in diameter) and exosomes (~30–150 nm in diameter) [33,34,35]. The nomenclature of EVs, based on the size of EVs, are referred to also by small EVs (<200 nm) or medium/large EVs (>200 nm) [36]. Small EVs, especially exosomes, are microvesicles with an endosomal origin which are formed by the internal budding of the multivesicular body membrane. Their presence in the extracellular area was first identified as early as the late 1980s [37]. It was proven that EVs/exosomes are produced and secreted from various cell types and occur in almost all kinds of bodily fluids. The characteristics of secreted EVs/exosomes are highly dependent on the origin, type and condition of parent cells. Presently, EVs/exosomes have evoked high attention and have been implicated both in physiological and pathological conditions. MSCs-derived exosomes are considered as special agents of intracellular communication and play an important role in tissue repair. Current studies indicate that MSCs-derived exosomes have similar biological activities and therapeutic outcomes when compared to MSCs alone [38,39,40] and are considered to be a good alternative to cell therapy.

**Figure 1:**
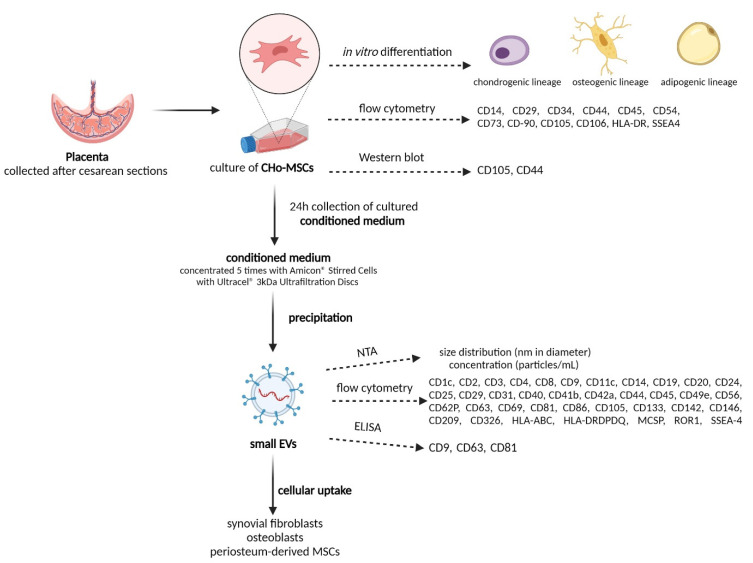
Schematic summary of CHo-MSCs cultivation, sEVs isolation and their downstream analyses (created with BioRender.com).

The proper definition and characterization of the terms EVs and exosomes varies in the literature. For this reason and regarding their determined characteristics, in this work we call them, collectively, small EVs (sEVs). Our study focused on the characterization of MSCs isolated from the chorion of human full-term placenta, as well as on the isolation and analysis of sEVs derived from the conditioned medium of CHo-MSCs under defined conditions. The aim of this study was to determine the surface markers and multilineage differentiation ability (adipogenic, osteogenic and chondrogenic induction) of CHo-MSCs expanded in vitro. sEVs were isolated using the precipitation method and characterized in terms of their size, yield and surface marker expression. The work-flow of our experiments is indicated on Figure 1. Finally, we demonstrated the ability of sEVs’ uptake by synovial fibroblasts, osteoblasts and periosteum-derived MSCs.

## 2. Results and Discussion

### 2.1. The Identification of Human Chorionic-Derived MSCs

**Figure 2:**
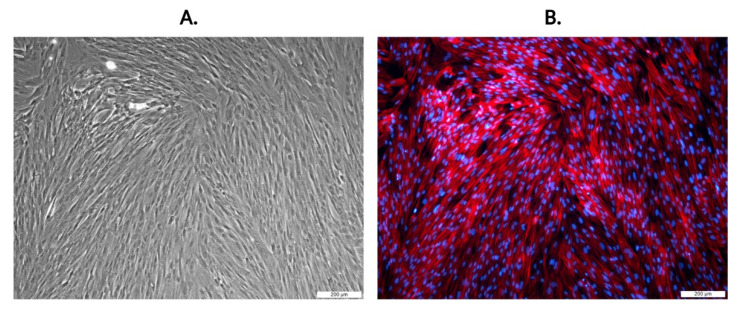
Morphology of CHo-MSCs in passage P4. (**A**) Bright-field (**B**) CHo-MSCs were stained with phalloidin (TRITC-conjugated fluorophore that labels actin microfilaments, red) and DAPI (fluorophore that labels nuclei, blue). The samples were imaged on an inverted fluorescence microscope Leica DMI3000B using a 10× objective. Scale bars = 200 µm.

**Figure 3:**
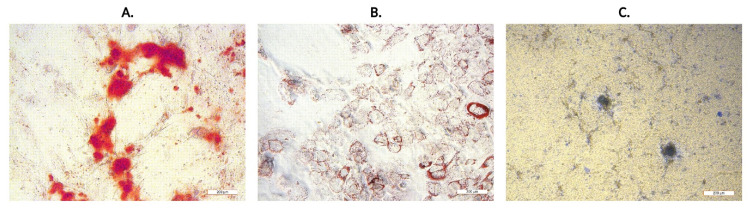
Microscopic images of CHo-MSCs (passage P4) after 21 days in differentiation medium. (**A**) Osteogenic differentiation detected by Alizarin Red S staining of calcium oxalates; (**B**) Adipogenic differentiation detected by Oil Red O staining of lipid vacuoles; (**C**) Chondrogenic differentiation detected by Alcain Blue staining of proteoglycans in differentiated human CHo-MSCs. Images were obtained on an inverted fluorescence microscope Leica DMI3000B using a 10× objective. Scale bars = 200 µm.

**Figure 4:**
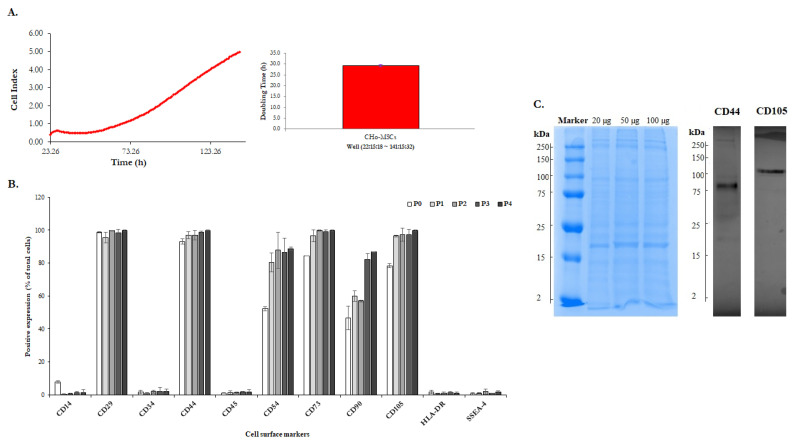
(**A**) Proliferation growth curve and doubling time of CHo-MSCs monitored by the xCELLigence^®^ RTCA SP system in real time. (**B**) Bar chart of flow cytometry data showing cell surface marker expression of human CHo-MSCs at different passages (P0–P4); *n* = 4. Values ± SD are shown as the percentage positive expression of total cells analyzed using flow cytometry. Positivity for each antibody was defined as the level of fluorescence greater than 95% of the isotype-matched control antibodies and a negativity less than 2%. (**C**) SDS-PAGE (20, 50 and 100 µg CHo-MSCs lysates were separated on 4–10% SDS-PAGE gel) with following Coomassie Blue staining (left, blue) and representative images from Western Blot analysis of MSCs- specific CD44 and CD105 proteins (right, gray). 50 µg CHo-MSCs lysates were electrophoretically transferred to nitrocellulose membrane and incubated with rabbit monoclonal anti-CD105 and anti-CD44 primary antibodies.

The placenta is considered as medical waste and is discarded after childbirth. Nevertheless, this fetal tissue is often described as a good and easily available source of MSCs for stem cell therapy, transplantation and tissue regeneration [11,41,42,43,44]. Originally, the placenta evolves from cells of fetal origin and progressively includes both maternal tissue (decidua) and fetal tissue (chorion, amnion). A predominant fraction of the placental external membrane surrounding the fetus is chorion, which has been shown to be a source of high amount of MSCs, with potential therapeutic effects [45]. There are three minimum criteria for MSCs [46]: being plastic adherent cells; having a differentiation potential to one or more lineages—adipogenic, osteogenic, chondrogenic or vascular/endothelial; being positive for the surface antigens CD90^+^, CD73^+^, CD105^+^ and negative for CD45^−^, CD34^−^, CD14^−^ and HLA-DR^−^. The isolated cells were characterized for their ability to adhere, differentiate into three mesodermal lineages and to express typical mesenchymal stromal cell markers. In the early stages of CHo-MSCs culture, cells adhered to the surface of tissue culture plates and reached 80–90% confluence after ~14 days with the cell doubling time 29.3 ± 0.5 h (Figure 4A). CHo-MSCs changed from small spindle-shaped cells to a fibroblast-like cellular morphology population during cultivation (Figure 2). Figure 2B is a representative image of fluorescently stained actin cytoskeleton of CHo-MSCs in passage four. It was observed that a large number of thin, parallel microfilament bundles extended across the entire cytoplasm in the CHo-MSCs’ actin cytoskeleton. Recent studies are focused on MSCs´ potential to differentiate into multiple tissue types—adipocytes [47], myocytes [48], osteocytes [49], hepatocytes [50] and neurons [51]—and have also suggested that there are differences in their degree of differentiation between source material and its age. To investigate whether isolated CHo-MSCs are able to differentiate into three lineages, chondrogenic, osteogenic and adipogenic, differentiation assays were induced in vitro. Osteogenic differentiation was proven by Alizarin Red S staining, where calcium oxalate deposits were detected (Figure 3A) in differentiated CHo-MSCs. Adipogenic differentiation was determined by Oil Red O staining of lipid vacuoles (Figure 3B). Chondrogenic differentiation was confirmed by Alcain Blue staining of proteoglycans (Figure 3C). The acquired results confirmed the nature of our isolated chorionic-derived cells as MSCs and showed their three-lineage differentiation capability.

For the confirmation of the mesenchymal character of the isolated cells, their phenotype was analyzed by flow cytometry. CHo-MSCs expressed multiple markers of MSCs. The percentage of positivity of each marker in passages P0–P4 is shown in Figure 4B. Flow cytometric analysis clearly indicated that CHo-MSCs at various passages were highly positive for all MSCs-specific surface markers (CD29, CD 44, CD73, CD90, CD105) and these expressions slightly increased with increasing passages. The expression of CD54 (known as ICAM-1, which is typically expressed on endothelial cells and cells of the immune system) also increased during the tested passages. On the other hand, the expression of hematopoietic marker (CD34), leukocyte marker (CD45), monocyte/macrophage marker (CD14) and embryonic stem cell marker (SSEA4) were negative at all tested passages. CHo-MSCs with higher passages also demonstrated a lower expression of major histocompatibility complex (MHC) class II HLA-DR when compared with P0.

The total protein content of CHo-MSCs lysates was ~1.82 mg/mL according to the results of BCA assay. In order to visualize and quantify the protein content in CHo-MSCs lysates, SDS-PAGE electrophoresis followed by Coomasie Blue staining was performed. In Figure 4C (on the left, blue) it is shown that a broad spectrum of proteins was detected in CHo-MSCs lysates and that each fraction showed a characteristic size distribution profile in the range of 2 to 250 kDa by 20, 50 and 100 µg of total proteins in lysates. Notably, in Figure 4C (on the right, grey) Western Blot analysis indicated that MSC-associated proteins, such as CD44 (band size at 80 kDa) and CD105 (band size at 105 kDa) were also detected in CHo-MSCs lysates.

Obtained data confirmed that the isolated CHo-MSCs in this study consistently fulfilled the criteria defined for MSCs [13,46] and that these cells can be used in our next experiment, which is focused on the isolation and characterization of sEVs. CHo-MSCs were expanded for seven passages with no significant changes in their phenotype characteristics. Therefore, we decided to use CHo-MSCs from P4 for sEVs isolation.

### 2.2. Isolation and Characterization of sEVs

Recent studies have reported various technical standardized tools and methods for the consistent isolation of high-yield and high-purity EVs [52] which could be used for further research of their composition, function and mechanism of action. EVs can be specifically isolated from a broad spectrum of cellular debris, interfering components and human samples. Frequently used methods for the isolation of EVs involve, among others, density gradient ultracentrifugation, differential ultracentrifugation, ultrafiltration, precipitation, size exclusion chromatography, immunoaffinity isolation and field-flow fractionation [53]. Each procedure has its advantages and disadvantages and may be exchanged, supplemented or combined with each other according to the sample source and intended use of EVs. It is apparent that EVs isolated from biofluids are characterized by a mixed cellular origin. Therefore, we decided to analyze sEVs obtained from a single cell type by collecting MSCs-conditioned medium (MSCs-CM) from cultured CHo-MSCs. MSCs-CM was concentrated using Ultracel^®^ 3 kDa Ultrafiltration Discs. During the procedure, gas pressure was applied directly to the Amicon^®^ Stired Cell. It is known that a solution larger than the membrane´s pore size is retained in the cell, while water and samples smaller than the pore size pass through the membrane into the filtrate.

In this work, five times concentrated MSCs-CM (retentate solution) was used for sEVs isolation by the precipitation method using ExoQuick reagent. The main objective of the precipitation method is to capture EVs by incubating with polymers which enable the acquirement of EVs at low speeds of centrifugation in combination with polymers [54]. This method is convenient, technically accessible, not time consuming and does not require a large sample ”starting“ volume. Serrano-Pertierra et al. indicated that precipitation reagents were more effective and resulted in larger numbers of EVs factors in comparison with ultracentrifugation [55]. Coughlan et al. also detected that the precipitation method for exosomes’ isolation was six times faster and led to the production of a 2.5-fold higher concentration of exosomes when compared with ultracentrifugation [56]. Accordingly, compared to other studies, when conditioned medium [57], plasma [58] or serum [57,59] have been used as a source of EVs, similar particle size and concentrations were shown for ExoQuick precipitation.

**Figure 5:**
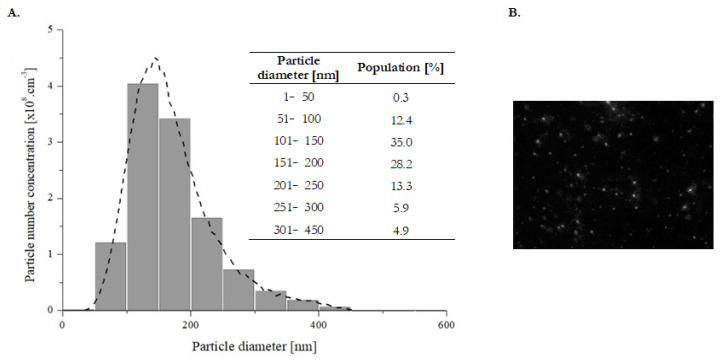
(**A**) Particle size distribution and percentage in various size ranges; (**B**) Representative capture of the corresponding video of isolated sEVs from concentrated MSCs-CM obtained by NTA (*n* = 4). The captured area of the microscopic image was 80 × 100 μm while the focal depth was approximately 20 μm.

In order to characterize and identify isolated sEVs, we used multiple approaches according to the ISEV [60]. To identify whether isolated EVs included mostly sEVs or a mixture of different EV types, several methods for their characterization were performed. The number and the size distribution of sEVs in prepared samples were evaluated by NTA, which is based on the measurement of Brownian motion of particles. Information about particles in solution is determined by capturing the tracks of particles that have scattered light upon illumination with a laser, calculating diffusion coefficients from the tracks, and subsequently calculating sphere-equivalent hydrodynamic radii from diffusion coefficients using the Stokes-Einstein equation [61]. Size distribution profiles were averaged within each sample (*n* = 4) across the video replicates, following the average across samples, to provide representative size distribution profiles. Particles in our samples were typically polydisperse, with a size distribution ranging from 50 to 400 nm in diameter. NTA showed that the majority of isolated EVs had a similar size of 169.2 ± 11.6 nm. Regarding the concentration of isolated samples, particle concentration was evaluated as 1.19 ± 0.88 × 10^9^ particles/mL (Figure 5A). In general, it is accepted that the size of EVs with endocytic origin (commonly known as sEVs or exosomes) is typically in the range of ~30–150 nm in diameter [33,35]. Since 47.4% of all isolated particles were in the typical sEVs size range, our results indicate that our samples, isolated from concentrated MSCs-CM, are rich in sEVs.

**Figure 6:**
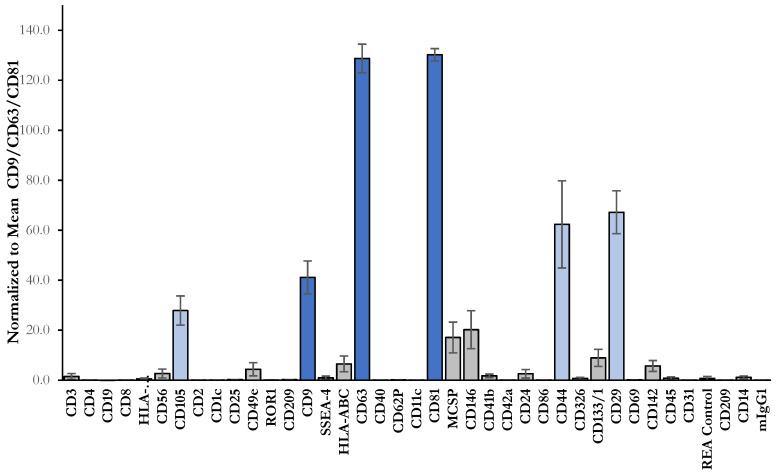
Phenotype of isolated MSCs-CM-derived sEVs by MACSPlex analysis. Data were normalized to mean CD9/CD63/CD81 and displayed in MACSPlex analysis of sEVs (*n* = 4) ± SD.

Exosomes are also defined based on their protein content, confirming their endosomal origin. Composition of exosomes includes proteins participating in membrane transport, fusion (e.g., annexins, flotillin, GTPases) and in multivesicular bodies’ biogenesis (e.g., ALIX, TSG101), as well as that of heat shock proteins (HSP70, HSP90) and also integrins and tetraspanins (CD9, CD63, CD81 and CD82) [62,63,64]. Tetraspanin proteins such as CD9, CD63 and CD81 with exposed domains, are especially enriched in the exosomal membrane and are considered as specific biomarkers of exosomes [65]. sEVs isolated from MSCs-CM were characterized using the commercial multiplex bead-based analysis (MACSPlex Exosome Kit) by flow cytometry. This platform allows for the detection of 37 exosomal surface epitopes and two isotype controls. In our study, typical exosomal markers (CD9, CD63 and CD81) and markers of the cell origin (CD29, CD44 and CD105) were detected. Isolated sEVs also expressed CD146 and MCSP (pericyte markers), but were negative for others’ epitopes. The highest expression was observed for the markers CD63 and CD81 (Figure 6). Furthermore, the quantification of sEVs isolated from MSCs-CM with ExoELISA-ULTRA showed that sEVs’ abundance in CD63 and CD81 was 1.51 ± 0.14 × 10^10^ and 5.92 ± 0.41 × 10^9^, respectively. Double sandwich ELISA confirmed the presence of CD9 at 1.01 ± 0.05 × 10^6^ (Table 1). The detection of all three exosomal markers in our samples confirmed the presence of sEVs, which is in accordance with the results of Damania et al. They characterized exosomes in a fractionated MSCs-CM secretome based on the presence of endosomal membrane markers CD9 (by sandwich ELISA), CD63 (by flow cytometry) and CD81 (by Western Blot) [66]. Likewise, Garcia-Contreras et al. detected the presence of plasma-derived exosomes with a surface expression of CD9, CD63 and CD81 via flow analysis [67].

### 2.3. Cellular Uptake of sEVs

**Figure 7:**
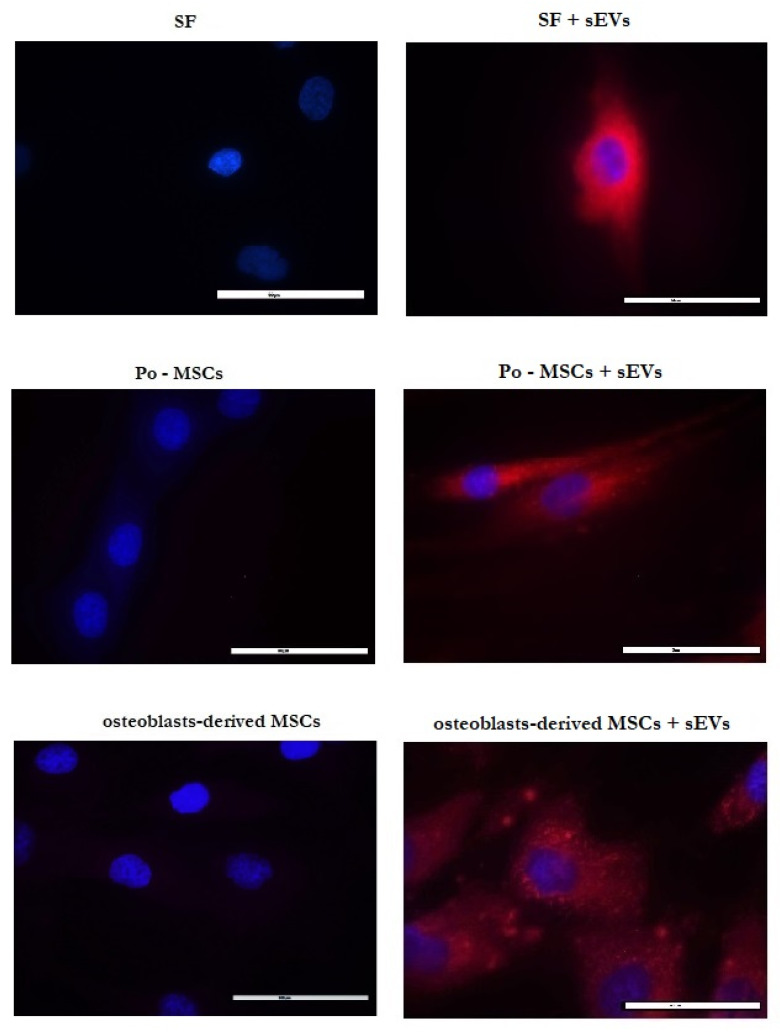
Fluorescence microscopy images demonstrated uptake of MSCs-CM-derived sEVs (labeled with ExoGlow MembraneT^M^ labeling dye, red) by SF, Po-MSCs and osteoblasts (cell nuclei stained with DAPI, blue). Cells treated with PBS without sEVs were used as controls. Scale bar = 50 μm.

The ability of exosomes to transpose composition from donor to target cells belongs to the interesting approaches used for cell-to-cell communication, with which not only physiological but also pathological signals are exchanged, mostly via transfer of bioactive proteins, lipids, RNAs and miRNAs [68]. The biologically active cargo of exosomes is able to alter gene expression and modulate the activity, function and composition of target cells [69]. Internalization of exosomes into the cells is considered to be one of the mechanisms of cargo delivery to recipient cells and, as such, affects their fate [70,71], however, the precise mechanism of these proceedings is not well understood and is still under examination. Currently it is known that exosomal content is connected to the therapeutic effect of MSCs-derived exosomes, most evident in the repair and regeneration of injured tissue during the osteoarthritic process (OA) [72,73]. Specifically, exosomes derived from different types of MSCs can protect the OA joint from damage by supporting cartilage repair, inhibiting synovitis and mediating subchondral bone remodeling, as well as have been demonstrated to regulate cartilage regeneration and to attenuate OA progression in certain models [27,72,74,75,76,77]. Therefore, three types of cells isolated from tissues mostly associated with OA were selected in this work in order to determine the uptake of MSCs-CM-derived sEVs by these recipient cells. Isolated sEVs were labeled with ExoGlow Membrane^TM^ labeling dye and co-cultured with OA-associated synovial fibroblasts (SF), periosteum-derived MSCs (Po-MSCs) and osteoblasts in vitro. As it is shown in Figure 7, sEVs (red fluorescence) were internalized and accumulated into cytoplasm and around the nuclei of all types of treated cells after 24 h incubation when compared with control (cells treated with PBS without sEVs). Results confirmed that sEVs isolated from CHo-MSCs could be taken up by all tested cell types, suggesting their direct interaction and potential relevance in cell-to-cell communication in the sEVs-based treatment of OA. However, for deeper understanding of the mechanism of sEVs uptake and their subsequent cargo delivery into the cytosol, further investigations are needed. From the existing evidence and obtained findings we can conclude that sEVs might be a promising tool for treatment strategies which exclude cells.

## 3. Materials and Methods

### 3.1. Tissue Collection and Isolation of Cells

Tissue harvesting and isolation of human cells was in accordance with ethical approval of the Louis Pasteur University Hospital in Kosice, Slovakia and realized after obtaining informed consent.

Placentas from healthy donor mothers (*n* = 4) were collected after cesarean sections. Before the placental tissue was separated, the fresh placenta was preserved in transport medium (DMEM (Sigma Aldrich, Steinheim, Germany) with 80 µg/mL gentamicin (Sigma Aldrich, Steinheim, Germany). Part of the chorion (10 × 10 cm) was dissected manually and washed intensively in PBS containing 100 IU penicillin/mL, 100 μg streptomycin/mL and 0.25 μg amphotericin B/mL (1% (*v*/*v*) antibiotic/antimycotic solution) (Sigma Aldrich, Steinheim, Germany) to remove red blood cells. Subsequently, the chorion was cut into small pieces (1.5 × 1.5 cm) and treated for 20 min with 2.4 U/mL dispase I solution (Gibco, Bleiswijk, The Netherlands) in DMEM containing 1% (*v*/*v*) antibiotic/antimycotic solution (Sigma Aldrich, Steinheim, Germany) at 37 °C. After incubation, the tissue sample was properly vortexed and then washed in DMEM and centrifuged at 300× *g* for 15 min. Then, chorion fragments were digested with 1.0 mg/mL collagenase type II at 37 °C for 2 h. Digested chorion fragments were washed again with DMEM, centrifuged at 300× *g* for 15 min and passed through a 40 µm cell strainer (BD Falcon™, Biosciences, New Jersey, USA). The cell suspension was washed two times with DMEM and cells were collected by centrifugation at 300× *g* for 15 min. The obtained chorionic (CHo) cells were cultured in a complete cultivation medium, α-MEM (Sigma Aldrich, Steinheim, Germany), supplemented with 10% FBS (Gibco, Bleiswijk, The Netherlands), 1% (*v*/*v*) antibiotic/antimycotic solution and 1% L-glutamine (Sigma Aldrich, Steinheim, Germany). CHo-MSCs were maintained in 75-cm^2^ culture flasks (Sarstedt AG & Co., Nümbrecht, Germany) at 37 °C, 95% humidity and in a 5% CO_2_ atmosphere. Non-attached cells were removed after three to five days of incubation. The cultivation medium was changed twice a week. When the cells reached 80% confluence, they were detached from the flask by 0.05% Trypsin-EDTA solution (Gibco, Bleiswijk, The Netherlands) for 2 min at 37 °C and seeded at a density of 2 × 10^3^ cells/cm^2^ (first passage). The number and viability of cells were assessed by a TC10™ Automated Cell Counter (Bio-Rad Laboratories, Hercules, CA, USA). The cells were expanded for 7 passages. Expression of surface biomarkers was monitored after every passage by flow cytometry. The cells were also seeded onto a 6-well plate in order to stain actin microfilaments and nuclei with phalloidin and DAPI, respectively.

Human synovium, periosteum and cancellous bone were obtained from donors who had undergone total knee replacement surgery due to osteoarthritis of the knee joint. SF and Po-MSCs were isolated by enzymatic digestion of the appropriate tissue [9]. Osteoblasts were isolated from cancellous bone [78]. Digested cells were filtered through a 40 μm cell strainer (BD Falcon™, Biosciences, New Jersey, USA) and the remaining tissues were discarded. Nucleated cells were plated on culture flasks (Sarstedt AG & Co., Nümbrecht Germany) and cultured in complete cultivation medium, α-MEM (Sigma Aldrich, Steinheim, Germany) supplemented with 10% FBS (Gibco, Bleiswijk, The Netherlands), 1% (*v*/*v*) antibiotic/antimycotic solution and 1% L-glutamine (Sigma Aldrich, Steinheim, Germany). Cells from passage 2 were used in further experiments.

### 3.2. In Vitro Differentiation

The potential of CHo-MSCs to differentiate into chondrogenic, osteogenic and adipogenic lineages was examined using the following procedures. Cells at a density of 1 × 10^5^ were seeded into each well of a 24 well tissue culture plate for differentiation. When the cells reached 60–80% confluence, an appropriate induction differentiation kit (StemPro Differentiation Kit; Gibco, Bleiswijk, The Netherlands) was used for differentiating the cells, according to the manufacturer’s instructions. After 2–3 weeks, cells were stained with Alizarin Red S (Sigma Aldrich, Steinheim, Germany) to detect calcium deposits and Alcian Blue (Merck, Kenilworth, NJ, USA) for evaluating chondrogenic differentiation. Oil Red O (Sigma Aldrich, Steinheim, Germany) staining was used for the evaluation of intracellular lipid accumulation. For the control group, 1 × 10^5^ cells were cultured with common culture media (α-MEM, 10% FBS, 1% ATB) without differentiation agents, and the same procedure of staining was performed.

### 3.3. Phenotypic Characterization of CHo-MSCs

Flow cytometry analysis of the expression of cell surface markers were performed on CHo-MSCs at passage P0–P7. The cells were harvested and centrifuged at 300× *g* for 10 min and washed with 1 × PBS containing 2% FBS. A minimum of 2 × 10^5^ cells were incubated with either fluorescein isothiocyanate (FITC)-, phycoerythrin (PE)- or allophycocyanin (APC)-conjugated antibodies: CD14, CD29, CD34, CD44, CD45, CD54, CD73, CD-90, CD105, HLA-DR, and SSEA4 for 10 min in the dark, and were then washed and centrifuged at 300× *g* for 10 min. The resuspended cell pellets were analyzed with a Becton Dickinson FACSCalibur using CellQuestPro software (Becton Dickinson).

### 3.4. Proliferation Assay in Real Time

For monitoring of the proliferation of CHo-MSCs in real time, the xCELLigence^®^ RTCA SP system was used. The procedure was carried out as previously described in our in vitro study [79]. Briefly, cells were seeded in a 96 well microtiter E-Plate^®^ (3000 cells per well) and the impedance value of each well was automatically monitored every hour and expressed as the cell index (CI) during 5 days. Proliferation curves and doubling time were analyzed by RTCA software.

### 3.5. Preparation of Protein Lysate from Cell Culture

When CHo-MSCs reached 80% confluence, the cultivation medium was discarded, and cells were washed with ice-cold PBS. Next, ice-cold lysis RIPA buffer was added, and cells were scraped using a cold plastic cell scraper and collected into pre-cooled microfuge tubes. The samples were agitated for 30 min at 4 °C and then centrifuged at 16,000× *g* for 20 min. Acquired supernatant was collected into fresh tubes on ice and stored at −80 °C before experiments.

### 3.6. Western Blot Analysis

Equal protein amount of each sample was mixed with 4× Laemmli-buffer in a ratio of 4:1 and boiled for 5 min at 95 °C. Samples were then separated on 4–10% SDS-Page gels and transferred to nitrocellulose membrane (Bio-Rad Laboratories, Hercules, CA, USA). The membrane was blocked in 5% skimmed milk (Sigma Aldrich, Steinheim, Germany) in TBS-Tween for 1 h at RT. The membrane, loaded with CHo-MSCs, was incubated with rabbit monoclonal anti-CD105 and anti-CD44 (Abcam, Germany), overnight at 4 °C with gentle rolling. Secondary antibody, goat anti-rabbit IgG H&L HRP-conjugated (Abcam, Germany), was used at 1:1000 for 1 h incubation at 4 °C with gentle rolling. Blots were shortly submerged into 3,3′,5,5′-Tetramethylbenzidine (TMB) substrate and immediately visualized by using the GelDoc EZ Imager (Bio-Rad Laboratories, Hercules, CA, USA).

### 3.7. Preparation of CHo-MSCs Conditioned Medium

When CHo-MSCs reached 80% confluence, the cells were washed with sterile PBS and the complete cultivation medium was replaced by DMEM without Phenol Red (Sigma Aldrich, Steinheim, Germany). The cell culture medium was collected after an additional 24 h incubation, centrifuged at 300× *g* for 10 min and subsequently filtered through a 0.22 µm filter. The collected medium was defined as MSCs-conditioned medium (MSCs-CM). CM was then concentrated 5 times using Amicon^®^ Stirred Cells with Ultracel^®^ 3 kDa Ultrafiltration Discs (Merck Life Science, USA) and aliquots were stored at −80 °C before use.

### 3.8. Isolation of sEVs

sEVs were isolated from 5 times concentrated MSCs-CM after the preliminary removal of cellular debris and large vesicles by centrifugation (3000× *g* for 15 min) with the Exosome Precipitation Kit (System Biosciences, Palo Alto, Canada), in accordance with the manufacturer’s recommendations. Briefly, prepared MSCs-CM was mixed with precipitation solution ExoQuick-TC (10 mL of MSCs-CM/2 mL of ExoQuickTC) and incubated overnight at 4 °C. After incubation, ExoQuick-TC/MSCs-CM mixtures were centrifuged at 1500× *g* for 30 min. Precipitated sEVs were diluted into sterile PBS and stored at −20 °C before the following experiments.

### 3.9. Assessment of Total Protein Content

The total protein concentrations from the CHo-MSCs and isolated sEVs were evaluated by a Rapid Gold bicinchoninic acid (BCA) assay kit (Thermo Scientific, Waltham, USA), in accordance with the manufacturer’s recommendations, and analyzed on a multimode reader (TRISTAR, Berthold Technologies). The global protein content was analyzed by SDS-Page, followed by Coomassie Blue staining.

### 3.10. Multiplex Bead-Based Flow Cytometry Analysis of the sEVs

Isolated sEVs were characterized by flow cytometry using the MACSPlex Exosome Kit (Miltenyi Biotec, Bergisch Gladbach, Germany), which was developed for the simultaneous detection of 37 surface epitopes (CD1c, CD2, CD3, CD4, CD8, CD9, CD11c, CD14, CD19, CD20, CD24, CD25, CD29, CD31, CD40, CD41b, CD42a, CD44, CD45, CD49e, CD56, CD62P, CD63, CD69, CD81, CD86, CD105, CD133, CD142, CD146, CD209, CD326, HLA-ABC, HLA-DRDPDQ, MCSP, ROR1 and SSEA-4) that are known to be present on different exosomes and two isotype control beads. Isolated sEVs were incubated with MACSPlex Exosome Capture Beads overnight in the dark on an orbital shaker (450 rpm) at RT. After incubation, sEVs were washed with MACSPlex buffer (MPB) and centrifuged at 3000× *g* for 5 min. Next, samples were incubated with APC-conjugated detection antibodies for 1 h at RT in the dark on an orbital shaker (450 rpm). After washing, the APC signal intensity in each 39 specific bead populations was measured on a Becton Dickinson FACSCalibur using CellQuestPro software (Becton Dickinson). Median fluorescence intensities (MFI) for all the capture beads were corrected for background signal by subtracting the MFI values of each bead obtained from control sample (buffer only) from the MFI values of the respective beads incubated with sample. The measured MFI inside each gate of a separate bead population was normalized to the mean tetraspanin CD9/CD63/CD81 MFI values, in order to determine the relative levels of a surface marker. Results are listed as the average of 4 measurements ± SD.

### 3.11. Nanoparticle Tracking Analysis

Concentrations and size distributions of isolated sEVs were analyzed by Nanoparticle Tracking Analysis (NTA) on an LM10B Nanoparticle Characterization System from Nano Sight (Amesbury, U.K.), with a trinocular microscope and LM12 viewing unit containing a 60 mW laser working at λ = 405 nm. Samples were diluted 10 times in PBS (final volume of 1 mL) to obtain a particle concentration suitable for NTA measurements (between 1 × 10^7^ and 1 × 10^9^/mL) and were then analyzed. The vesicles moving under Brownian motion act as point scatters when illuminated with a laser beam. The light scattered by the vesicles is then captured using a video camera. Analysis of the video file allows one to track the motion of each vesicle in two dimensions on a frame-by-frame basis. The mean square displacement of the vesicles obtained from the analysis of the captured video is then used to calculate their diffusion coefficients and sphere-equivalent hydrodynamic radii via the Stokes-Einstein equation. Results are displayed as a number-weighted particle size distribution. Video sequences were recorded via a CCD camera operating at 30 frames per second (fps) and evaluated through the NANOSIGHT NTA 3.4 Analytical Software Suite. For each sample, five videos were recorded. The durations of the video sequences were selected based on the particle concentration in a specific sample.

### 3.12. Quantitation of sEVs Abundance Using ELISA Assays

ExoELISA-ULTRA (System Biosciences, Palo Alto, Canada) CD63 and CD81 kits and an ExoTEST (HansaBiomed, Tallinn, Estonia) double sandwich ELISA assay for CD9 were used to quantify the sEVs’ abundance in prepared samples, in accordance with the manufacturer’s recommendations. The absorbances were recorded at 450 nm by using a TriStar LB941 spectrophotometric plate reader (Berthold, Germany). Briefly, for ExoELISA-ULTRA: 50 µL of freshly prepared protein standards or sEV samples were added to a 96 well plate and incubated at 37 °C for 1 h by gentle shaking, followed by the adding of primary antibody (diluted in blocking buffer in ratios of 1:1000 and 1:100 for CD81 and CD63, respectively) and secondary antibody (1:5000 in blocking buffer) to each well for the next 1 h incubation at RT. Finally, super-sensitive TMB ELISA substrate was added to samples, the reaction was stopped with stop buffer and the absorbance was measured. For ExoTEST: 100 µL of prepared sEV samples were incubated on an immunoplate pre-coated with proprietary pan-exosome antibodies overnight at 37 °C in a humid chamber, followed by adding primary monoclonal biotin-conjugated anti-human CD9 antibody (1:500 in 1× sample buffer) and secondary streptavidin-HRP-conjugated antibody (1:5000 in 1× sample buffer). The reaction was developed with substrate chromogenic solution, blocked with stop solution and the absorbance was measured.

### 3.13. sEVs Uptake by Different Cells

Cells, which were isolated from three different tissues derived from osteoarthritis (OA) patients (SF, Po-MSCs and osteoblasts), were treated with labeled sEVs in PBS or with PBS without sEVs (control group) for 24 h. sEVs were labeled with SBI´s ExoGlow Membrane^TM^ EV Labeling Kit (System Biosciences, Palo Alto, Canada). It is the latest generation of fluorescent labeling reagent to robustly and specifically label the EV membranes. Briefly, isolated sEVs were added into the labeling reaction buffer, which consisted of reaction buffer and labeling dye, and incubated for 30 min at RT in the dark. For removing free unlabeled dye, the Exosome Spin Columns (MW 3000) (Invitrogen) were used. Potential sEV uptake by different cells was analyzed with a fluorescence microscope Nicon Eclipse Ti (Japan) directly after 24 h of incubation with labeled sEVs.

## 4. Conclusions

sEVs/exosomes, as important cell-to-cell communication factors, have shown powerful potential in the treatment of various diseases. Nonetheless, from pre-clinical studies of sEVs/exosomes therapy to the clinical application there are still many critical problems to be solved, including precise sEVs/exosome isolation protocols, their characterization, underlying mechanisms of action and diagnostic/therapeutic application. Our study showed the successful preparation and characterization of sEVs released by CHo-MSCs. Firstly, the criteria for MSCs isolated from the chorion of human full-term placenta were confirmed—they were able to differentiate into three lineages (chondrogenic, osteogenic and adipogenic differentiation); expressed multiple markers of MSCs and were plastic adherent. Directly, sEVs were isolated from the CM of CHo-MSCs by the precipitation method using ExoQuick. sEVs were confirmed in terms of their size and protein components. NTA analysis showed that 47.4% of isolated particles were in the typical sEVs/exosome range (30–150 nm). The presence of exosomal markers CD9, CD63 and CD81 in the samples was confirmed by flow cytometry and ELISA tests. Furthermore, the obtained results in this study also demonstrated that sEVs and their content can be uptaken by different types of cells isolated from tissues associated with OA (SF, osteoblasts and Po-derived MSCs) suggesting their perspective role in the treatment of osteoarthritis. Even though sEVs have a strong therapeutic potential, experiments based on the use of sEVs are still in their initial phase and further studies for better understanding how exosomal cargo can contribute to their biological activity need to be clarified before their future usage.

## Figures and Tables

**Table 1:** Detection of MSCs-CM-derived sEVs abundance.

Assay	Detection	sEVs Abundance
ELISA	CD9	1.01 ± 0.05 × 10^6^
	CD63	1.51 ± 0.14 × 10^10^
	CD81	5.92 ± 0.41 × 10^9^

## Data Availability

The study did not report any data.

## References

[1] Haynesworth S.E., Goshima J., Goldberg V.M., Caplan A.I. (1992). Characterization of cells with osteogenic potential from human marrow. Bone.

[2] Lazarus H.M., Haynesworth S.E., Gerson S.L., Rosenthal N.S., Caplan A.I. (1995). Ex-vivo expansion and subsequent infusion of human bone-marrow-derived stromal progenitor cells (mesenchymal progenitor cells)—Implications for therapeutic use. Bone Marrow Transplant..

[3] Pittenger M.F., Discher D.E., Peault B.M., Phinney D.G., Hare J.M., Caplan A.I. (2019). Mesenchymal stem cell perspective: Cell biology to clinical progress. NPJ Regen. Med..

[4] Spakova T., Plsikova J., Harvanova D., Lacko M., Stolfa S., Rosocha J. (2018). Influence of Kartogenin on Chondrogenic Differentiation of Human Bone Marrow-Derived MSCs in 2D Culture and in Co-Cultivation with OA Osteochondral Explant. Molecules.

[5] Hunakova K., Hluchy M., Spakova T., Matejova J., Mudronova D., Kuricova M., Rosocha J., Ledecky V. (2020). Study of bilateral elbow joint osteoarthritis treatment using conditioned medium from allogeneic adipose tissue-derived MSCs in Labrador retrievers. Res. Vet. Sci..

[6] Savkovic V., Li H., Seon J.K., Hacker M., Franz S., Simon J.C. (2014). Mesenchymal stem cells in cartilage regeneration. Curr. Stem Cell Res. Ther..

[7] Murphy M.B., Moncivais K., Caplan A.I. (2013). Mesenchymal stem cells: Environmentally responsive therapeutics for regenerative medicine. Exp. Mol. Med..

[8] Franz J., Catarina L., Tatjana S., Peggy B., Lothar S., Regina E. (2008). Biology of Mesenchymal Stem Cells. Curr. Rheumatol. Rev..

[9] Harvanova D., Tothova T., Sarissky M., Amrichova J., Rosocha J. (2011). Isolation and Characterization of Synovial Mesenchymal Stem Cells. Folia Biol..

[10] Pountos I., Giannoudis P.V. (2005). Biology of mesenchymal stem cells. Inj. Int. J. Care Inj..

[11] Heeger P.S. (2004). Amnion and chorion cells as therapeutic agents for transplantation and tissue regeneration: A field in its infancy. Transplantation.

[12] Choi Y.S., Park Y.B., Ha C.W., Kim J.A., Heo J.C., Han W.J., Oh S.Y., Choi S.J. (2017). Different characteristics of mesenchymal stem cells isolated from different layers of full term placenta. PLoS ONE.

[13] Parolini O., Alviano F., Bagnara G.P., Bilic G., Bühring H.J., Evangelista M., Hennerbichler S., Liu B., Magatti M., Mao N. (2008). Concise review: Isolation and characterization of cells from human term placenta: Outcome of the first international Workshop on Placenta Derived Stem Cells. Stem Cells.

[14] Bačenková D., Rosocha J., Tóthová T., Rosocha L., Šarisský M. (2011). Isolation and basic characterization of human term amnion and chorion mesenchymal stromal cells. Cytotherapy.

[15] Gonzalez P.L., Carvajal C., Cuenca J., Alcayaga-Miranda F., Figueroa F.E., Bartolucci J., Salazar-Aravena L., Khoury M. (2015). Chorion Mesenchymal Stem Cells Show Superior Differentiation, Immunosuppressive, and Angiogenic Potentials in Comparison with Haploidentical Maternal Placental Cells. Stem Cells Transl. Med..

[16] Bačenková D., Trebuňová M., Zachar L., Hudák R., Ižaríková G., Šurínová K., Živčák J. (2020). Analysis of Same Selected Immunomodulatory Properties of Chorionic Mesenchymal Stem Cells. Appl. Sci..

[17] Koo B.K., Park I.Y., Kim J., Kim J.H., Kwon A., Kim M., Kim Y., Shin J.C., Kim J.H. (2012). Isolation and Characterization of Chorionic Mesenchymal Stromal Cells from Human Full Term Placenta. J. Korean Med. Sci..

[18] Yamahara K., Harada K., Ohshima M., Ishikane S., Ohnishi S., Tsuda H., Otani K., Taguchi A., Soma T., Ogawa H. (2014). Comparison of Angiogenic, Cytoprotective, and Immunosuppressive Properties of Human Amnion- and Chorion-Derived Mesenchymal Stem Cells. PLoS ONE.

[19] Pittenger M.F., Mackay A.M., Beck S.C., Jaiswal R.K., Douglas R., Mosca J.D., Moorman M.A., Simonetti D.W., Craig S., Marshak D.R. (1999). Multilineage potential of adult human mesenchymal stem cells. Science.

[20] Haynesworth S.E., Baber M.A., Caplan A.I. (1996). Cytokine expression by human marrow-derived mesenchymal progenitor cells in vitro: Effects of dexamethasone and IL-1 alpha. J. Cell. Physiol..

[21] Akyurekli C., Le Y., Richardson R.B., Fergusson D., Tay J., Allan D.S. (2015). A systematic review of preclinical studies on the therapeutic potential of mesenchymal stromal cell-derived microvesicles. Stem Cell Rev. Rep..

[22] Janockova J., Slovinska L., Harvanova D., Spakova T., Rosocha J. (2021). New therapeutic approaches of mesenchymal stem cells-derived exosomes. J. Biomed. Sci..

[23] De Jong O.G., Van Balkom B.W.M., Schiffelers R.M., Bouten C.V.C., Verhaar M.C. (2014). Extracellular Vesicles: Potential Roles in Regenerative Medicine. Front. Immunol..

[24] Rong X., Liu J., Yao X., Jiang T., Wang Y., Xie F. (2019). Human bone marrow mesenchymal stem cells-derived exosomes alleviate liver fibrosis through the Wnt/β-catenin pathway. Stem Cell Res. Ther..

[25] Zhang Y.L., Chopp M., Zhang Z.G., Katakowski M., Xin H.Q., Qu C.S., Ali M., Mahmood A., Xiong Y. (2017). Systemic administration of cell-free exosomes generated by human bone marrow derived mesenchymal stem cells cultured under 2D and 3D conditions improves functional recovery in rats after traumatic brain injury. Neurochem. Int..

[26] Qin Y., Wang L., Gao Z., Chen G., Zhang C. (2016). Bone marrow stromal/stem cell-derived extracellular vesicles regulate osteoblast activity and differentiation in vitro and promote bone regeneration in vivo. Sci. Rep..

[27] Cosenza S., Ruiz M., Toupet K., Jorgensen C., Noël D. (2017). Mesenchymal stem cells derived exosomes and microparticles protect cartilage and bone from degradation in osteoarthritis. Sci. Rep..

[28] Zou X., Gu D., Xing X., Cheng Z., Gong D., Zhang G., Zhu Y. (2016). Human mesenchymal stromal cell-derived extracellular vesicles alleviate renal ischemic reperfusion injury and enhance angiogenesis in rats. Am. J. Transl. Res..

[29] Zou L.Y., Ma X.K., Lin S., Wu B.Y., Chen Y., Peng C.Q. (2019). Bone marrow mesenchymal stem cell-derived exosomes protect against myocardial infarction by promoting autophagy. Exp. Ther. Med..

[30] Cui G.H., Wu J., Mou F.F., Xie W.H., Wang F.B., Wang Q.L., Fang J., Xu Y.W., Dong Y.R., Liu J.R. (2018). Exosomes derived from hypoxia-preconditioned mesenchymal stromal cells ameliorate cognitive decline by rescuing synaptic dysfunction and regulating inflammatory responses in APP/PS1 mice. FASEB J. Off. Publ. Fed. Am. Soc. Exp. Biol..

[31] Zhang B., Wu X., Zhang X., Sun Y., Yan Y., Shi H., Zhu Y., Wu L., Pan Z., Zhu W. (2015). Human umbilical cord mesenchymal stem cell exosomes enhance angiogenesis through the Wnt4/β-catenin pathway. Stem Cells Transl. Med..

[32] Théry C., Witwer K.W. (2018). Minimal information for studies of extracellular vesicles 2018 (MISEV2018): A position statement of the International Society for Extracellular Vesicles and update of the MISEV2014 guidelines. J. Extracell. Vesicles.

[33] Nederveen J.P., Warnier G., Di Carlo A., Nilsson M.I., Tarnopolsky M.A. (2021). Extracellular Vesicles and Exosomes: Insights from Exercise Science. Front. Physiol..

[34] Spakova T., Janockova J., Rosocha J. (2021). Characterization and Therapeutic Use of Extracellular Vesicles Derived from Platelets. Int. J. Mol. Sci..

[35] Doyle L.M., Wang M.Z. (2019). Overview of Extracellular Vesicles, Their Origin, Composition, Purpose, and Methods for Exosome Isolation and Analysis. Cells.

[36] Gould S.J., Raposo G. (2013). As we wait: Coping with an imperfect nomenclature for extracellular vesicles. J. Extracell. Vesicles.

[37] Johnstone R.M., Adam M., Hammond J.R., Orr L., Turbide C. (1987). Vesicle formation during reticulocyte maturation. Association of plasma membrane activities with released vesicles (exosomes). J. Biol. Chem..

[38] Heidari M., Pouya S., Baghaei K., Aghdaei H.A., Namaki S., Zali M.R., Hashemi S.M. (2018). The immunomodulatory effects of adipose-derived mesenchymal stem cells and mesenchymal stem cells-conditioned medium in chronic colitis. J. Cell. Physiol..

[39] Ma Z.J., Wang Y.H., Li Z.G., Wang Y., Li B.Y., Kang H.Y., Wu X.Y. (2019). Immunosuppressive Effect of Exosomes from Mesenchymal Stromal Cells in Defined Medium on Experimental Colitis. Int. J. Stem Cells.

[40] Reza-Zaldivar E.E., Hernández-Sapiéns M.A., Gutiérrez-Mercado Y.K., Sandoval-Ávila S., Gomez-Pinedo U., Márquez-Aguirre A.L., Vázquez-Méndez E., Padilla-Camberos E., Canales-Aguirre A.A. (2019). Mesenchymal stem cell-derived exosomes promote neurogenesis and cognitive function recovery in a mouse model of Alzheimer’s disease. Neural Regen. Res..

[41] Raynaud C.M., Maleki M., Lis R., Ahmed B., Al-Azwani I., Malek J., Safadi F.F., Rafii A. (2012). Comprehensive characterization of mesenchymal stem cells from human placenta and fetal membrane and their response to osteoactivin stimulation. Stem Cells Int..

[42] Lee J.M., Jung J., Lee H.J., Jeong S.J., Cho K.J., Hwang S.G., Kim G.J. (2012). Comparison of immunomodulatory effects of placenta mesenchymal stem cells with bone marrow and adipose mesenchymal stem cells. Int. Immunopharmacol..

[43] Wang L., Yang Y., Zhu Y., Ma X., Liu T., Zhang G., Fan H., Ma L., Jin Y., Yan X. (2012). Characterization of placenta-derived mesenchymal stem cells cultured in autologous human cord blood serum. Mol. Med. Rep..

[44] Parolini O., Alviano F., Bergwerf I., Boraschi D., De Bari C., De Waele P., Dominici M., Evangelista M., Falk W., Hennerbichler S. (2010). Toward cell therapy using placenta-derived cells: Disease mechanisms, cell biology, preclinical studies, and regulatory aspects at the round table. Stem Cells Dev..

[45] Nazarov I., Lee J.W., Soupene E., Etemad S., Knapik D., Green W., Bashkirova E., Fang X.H., Matthay M.A., Kuypers F.A. (2012). Multipotent Stromal Stem Cells from Human Placenta Demonstrate High Therapeutic Potential. Stem Cells Transl. Med..

[46] Dominici M., Le Blanc K., Mueller I., Slaper-Cortenbach I., Marini F., Krause D., Deans R., Keating A., Prockop D., Horwitz E. (2006). Minimal criteria for defining multipotent mesenchymal stromal cells. The International Society for Cellular Therapy position statement. Cytotherapy.

[47] Pfutzner A., Schipper D., Pansky A., Kleinfeld C., Roitzheim B., Tobiasch E. (2017). Mesenchymal Stem Cell Differentiation into Adipocytes Is Equally Induced by Insulin and Proinsulin In Vitro. Int. J. Stem Cells.

[48] Xie X.J., Wang J.A., Cao J., Zhang X. (2006). Differentiation of bone marrow mesenchymal stem cells induced by myocardial medium under hypoxic conditions. Acta Pharmacol. Sin..

[49] Hanna H., Mir L.M., Andre F.M. (2018). In vitro osteoblastic differentiation of mesenchymal stem cells generates cell layers with distinct properties. Stem Cell Res. Ther..

[50] Najimi M., Khuu D.N., Lysy P.A., Jazouli N., Abarca J., Sempoux C., Sokal E.M. (2007). Adult-derived human liver mesenchymal-like cells as a potential progenitor reservoir of hepatocytes?. Cell Transplant..

[51] Hernandez R., Jimenez-Luna C., Perales-Adan J., Perazzoli G., Melguizo C., Prados J. (2020). Differentiation of Human Mesenchymal Stem Cells towards Neuronal Lineage: Clinical Trials in Nervous System Disorders. Biomol. Ther..

[52] Yu L.L., Zhu J., Liu J.X., Jiang F., Ni W.K., Qu L.S., Ni R.Z., Lu C.H., Xiao M.B. (2018). A Comparison of Traditional and Novel Methods for the Separation of Exosomes from Human Samples. Biomed Res. Int..

[53] Witwer K.W., Buzas E.I., Bemis L.T., Bora A., Lasser C., Lotvall J., Hoen E.N.N., Piper M.G., Sivaraman S., Skog J. (2013). Standardization of sample collection, isolation and analysis methods in extracellular vesicle research. J. Extracell. Vesicles.

[54] Zhou M., Weber S.R., Zhao Y., Chen H., Sundstrom J.M., Edelstein L., Smythies J., Quesenberry P., Noble D. (2020). Chapter 2—Methods for exosome isolation and characterization. Exosomes.

[55] Serrano-Pertierra E., Oliveira-Rodríguez M. (2019). Characterization of Plasma-Derived Extracellular Vesicles Isolated by Different Methods: A Comparison Study. Bioengineering.

[56] Coughlan C., Bruce K.D., Burgy O., Boyd T.D., Michel C.R., Garcia-Perez J.E., Adame V., Anton P., Bettcher B.M., Chial H.J. (2020). Exosome Isolation by Ultracentrifugation and Precipitation and Techniques for Downstream Analyses. Curr. Protoc. Cell Biol..

[57] Tang Y.T., Huang Y.Y., Zheng L., Qin S.H., Xu X.P., An T.X., Xu Y., Wu Y.S., Hu X.M., Ping B.H. (2017). Comparison of isolation methods of exosomes and exosomal RNA from cell culture medium and serum. Int. J. Mol. Med..

[58] Stranska R., Gysbrechts L., Wouters J., Vermeersch P., Bloch K., Dierickx D., Andrei G., Snoeck R. (2018). Comparison of membrane affinity-based method with size-exclusion chromatography for isolation of exosome-like vesicles from human plasma. J. Transl. Med..

[59] Helwa I., Cai J., Drewry M.D., Zimmerman A., Dinkins M.B., Khaled M.L., Seremwe M., Dismuke W.M., Bieberich E., Stamer W.D. (2017). A Comparative Study of Serum Exosome Isolation Using Differential Ultracentrifugation and Three Commercial Reagents. PLoS ONE.

[60] Lötvall J., Hill A.F., Hochberg F., Buzás E.I., Di Vizio D., Gardiner C., Gho Y.S., Kurochkin I.V., Mathivanan S., Quesenberry P. (2014). Minimal experimental requirements for definition of extracellular vesicles and their functions: A position statement from the International Society for Extracellular Vesicles. J. Extracell. Vesicles.

[61] Bachurski D., Schuldner M., Nguyen P.H., Malz A., Reiners K.S., Grenzi P.C., Babatz F., Schauss A.C., Hansen H.P., Hallek M. (2019). Extracellular vesicle measurements with nanoparticle tracking analysis—An accuracy and repeatability comparison between NanoSight NS300 and ZetaView. J. Extracell. Vesicles.

[62] Raposo G., Stoorvogel W. (2013). Extracellular vesicles: Exosomes, microvesicles, and friends. J. Cell Biol..

[63] Théry C. (2011). Exosomes: Secreted vesicles and intercellular communications. F1000 Biol. Rep..

[64] Simpson R.J., Lim J.W., Moritz R.L., Mathivanan S. (2009). Exosomes: Proteomic insights and diagnostic potential. Expert Rev. Proteom..

[65] Andreu Z., Yáñez-Mó M. (2014). Tetraspanins in extracellular vesicle formation and function. Front. Immunol..

[66] Damania A., Jaiman D., Teotia A.K., Kumar A. (2018). Mesenchymal stromal cell-derived exosome-rich fractionated secretome confers a hepatoprotective effect in liver injury. Stem Cell Res. Ther..

[67] Garcia-Contreras M., Shah S.H., Tamayo A., Robbins P.D., Golberg R.B., Mendez A.J., Ricordi C. (2017). Plasma-derived exosome characterization reveals a distinct microRNA signature in long duration Type 1 diabetes. Sci. Rep..

[68] Lener T., Gimona M., Aigner L., Borger V., Buzas E., Camussi G., Chaput N., Chatterjee D., Court F.A., del Portillo H.A. (2015). Applying extracellular vesicles based therapeutics in clinical trials—An ISEV position paper. J. Extracell. Vesicles.

[69] Yuana Y., Sturk A., Nieuwland R. (2013). Extracellular vesicles in physiological and pathological conditions. Blood Rev..

[70] Wendler F., Bota-Rabassedas N., Franch-Marro X. (2013). Cancer becomes wasteful: Emerging roles of exosomes(†) in cell-fate determination. J. Extracell. Vesicles.

[71] Urbanelli L., Magini A., Buratta S., Brozzi A., Sagini K., Polchi A., Tancini B., Emiliani C. (2013). Signaling pathways in exosomes biogenesis, secretion and fate. Genes.

[72] Ni Z., Zhou S., Li S., Kuang L., Chen H., Luo X., Ouyang J., He M., Du X., Chen L. (2020). Exosomes: Roles and therapeutic potential in osteoarthritis. Bone Res..

[73] Lin J.J., Wang L., Lin J.H., Liu Q. (2021). The Role of Extracellular Vesicles in the Pathogenesis, Diagnosis, and Treatment of Osteoarthritis. Molecules.

[74] Tao S.-C., Yuan T., Zhang Y.-L., Yin W.-J., Guo S.-C., Zhang C.-Q. (2017). Exosomes derived from miR-140-5p-overexpressing human synovial mesenchymal stem cells enhance cartilage tissue regeneration and prevent osteoarthritis of the knee in a rat model. Theranostics.

[75] Woo C.H., Kim H.K., Jung G.Y., Jung Y.J., Lee K.S., Yun Y.E., Han J., Lee J., Kim W.S., Choi J.S. (2020). Small extracellular vesicles from human adipose-derived stem cells attenuate cartilage degeneration. J. Extracell. Vesicles.

[76] Liu Y., Zou R., Wang Z., Wen C., Zhang F., Lin F. (2018). Exosomal KLF3-AS1 from hMSCs promoted cartilage repair and chondrocyte proliferation in osteoarthritis. Biochem. J..

[77] Zhang S., Chu W.C., Lai R.C., Lim S.K., Hui J.H., Toh W.S. (2016). Exosomes derived from human embryonic mesenchymal stem cells promote osteochondral regeneration. Osteoarthr. Cartil..

[78] Harvanová D., Hornák S., Amrichová J., Spaková T., Mikes J., Plsíková J., Ledecký V., Rosocha J. (2014). Isolation, cultivation and characterisation of pigeon osteoblasts seeded on xenogeneic demineralised cancellous bone scaffold for bone grafting. Vet. Res. Commun..

[79] Amrichova J., Spakova T., Rosocha J., Harvanova D., Bacenkova D., Lacko M., Hornak S. (2014). Effect of PRP and PPP on proliferation and migration of human chondrocytes and synoviocytes in vitro. Cent. Eur. J. Biol..

